# Encapsulation of Vitamins Using Nanoliposome: Recent Advances and Perspectives

**DOI:** 10.34172/apb.2023.005

**Published:** 2021-10-10

**Authors:** Masoud Aman Mohammadi, Parastou Farshi, Parisa Ahmadi, Azam Ahmadi, Mohammad Yousefi, Marjan Ghorbani, Seyede Marzieh Hosseini

**Affiliations:** ^1^Student Research Committee, Department of Food Technology, Faculty of Nutrition Science and Food Technology, Nutritional and Food Technology Research Institute, Shahid Beheshti University of Medical Sciences, Tehran, Iran.; ^2^Food Science Institute, Kansas State University, Manhattan KS, USA.; ^3^Student Research Committee, Department of Food Sciences and Technology, Faculty of Nutrition and Food Sciences, Tabriz University of Medical Sciences, Tabriz, Iran.; ^4^Nutrition Research Center, Tabriz University of Medical Sciences, Tabriz, Iran.; ^5^Department of Food Technology, Faculty of Nutrition Sciences and Food Technology/National Nutrition and Food Technology Research Institute, Shahid Beheshti University of Medical Sciences, Tehran, Iran.

**Keywords:** Encapsulation, Vitamins, Nanoliposomes, Nanoliposome preparation, Nutraceuticals

## Abstract

Nowadays the importance of vitamins is clear for everyone. However, many patients are suffering from insufficient intake of vitamins. Incomplete intake of different vitamins from food sources due to their destruction during food processing or decrease in their bioavailability when mixing with other food materials, are factors resulting in vitamin deficiency in the body. Therefore, various lipid based nanocarriers such as nanoliposomes were developed to increase the bioavailability of bioactive compounds. Since the function of nanoliposomes containing vitamins on the body has a direct relationship with the quality of produced nanoliposomes, this review study was planned to investigate the several aspects of liposomal characteristics such as size, polydispersity index, zeta potential, and encapsulation efficiency on the quality of synthesized vitamin-loaded nanoliposomes.

## Introduction

 Vitamins are essential micronutrients considered as a class of organic compounds which are not synthesized in the human body (except vitamin D and B2), and are usually deprived of providing energy, however, they are extremely required for proper performance of body.^1^ There are thirteen vitamins which are categorized based on their solubility. Lipophilic vitamins are composed of A, E, K, and D vitamins along with carotenoids showing the functional traits of vitamin A, and hydrophilic vitamins are comprised of vitamin C and the group of B vitamins, including thiamin (B1), riboflavin (B2), nicotinic acid (B3), pyridoxine (B6), pantothenic acid (B5), folate, and cyanocobalamin (B12).^2^

 The importance of vitamins has been discussed in a separate section in this paper. In addition to the function of vitamins in the body, these micronutrients are able to prevent many diseases. The most important example that can be mentioned is COVID-19. Although, there are not adequate data regarding the function of vitamins against COVID-19, a certain number of recent studies have reported that some vitamins, particularly vitamin D may have an important role in improving the immune system of the body to act against the coronavirus.^3-6^

 In spite of many advantages of vitamins, they have poor bio-accessibility and bioavailability, and they are highly vulnerable to degradation. Different parameters during the process and storage may cause degradation of vitamins such as oxygen, temperature, light, water activity, moisture content, pH, metal trace elements especially iron and copper, and degradative enzymes (lipase, proteases, and nucleases).^7^ Encapsulation techniques can surround vitamins and protect them against sever conditions such as exposure to oxygen, heat, pro-oxidants, or UV light, throughout the storage and can also enhance their functional properties like solubility, stability and controlled release. Vitamin D and K have lipophilic nature with a week solubility in the aqueous media. To improve their stability, solubility, and targeting feature, some modifications are needed to be made.^8^ Thus, a wide range of delivery systems have been developed.

 Nanoliposome is considered as one of the lipid-based carriers, which is used for delivery of different bioactive compounds.^9^ Due to the presence of hydrophilic and hydrophobic components in the structure of nanoliposomes, they are considered as suitable encapsulants for loading and delivering different types of vitamins to target cells.^10^ Although, there are some studies related to the delivery of vitamins using lipid carriers such as the work of Hsu et al,^7^ the main purpose of this study is more detailed and comprehensive investigation of recent literature pertaining to the application and efficacy of nanoliposomes to encapsulate vitamins, especially in different foods and environmental conditions, is conducted.

## An overview of vitamins

 Vitamins are considered as one of the essential organic and micronutrient compounds with bioactive characteristics which are necessary for preserving the normal performance of human body through adjusting enzymatic and chemical reactions and physiological effects on various biological responses, including host immunity.^11,12^ Moreover, they play primary roles in different basic metabolic pathways, supporting the fundamental function of human cells. In particular, their participation in DNA synthesis, neuronal functions, energy-yielding metabolism, and oxygen transport makes them essential for muscular and brain functions. All of these factors can directly or indirectly affect the psychological and cognitive processes, including physical and mental fatigue.^1^ They can be extracted from food and supplements, or in some cases, synthesized by our body or gut microbiome.^13^

 Vitamins are classified into fat-soluble vitamins (vitamins A, D, E, and K) and water-soluble vitamins (vitamins B and C). Fat-soluble vitamins usually bind to cellular nuclear receptors and have an effect on the expression of particular genes.^14^ In addition, adequate amount of bile flow and micelle formation enhance the absorption of fat-soluble vitamins. In recent decades, insufficiencies of these vitamins have been the ground for expanded danger of tumor, type II diabetes mellitus and other various invulnerable framework issues.^15^ Water-soluble vitamins are mostly considered as cofactors for enzymes, influencing the enzymatic activity which can also improve the energy metabolism. Water-soluble vitamins are not stored in the body and any excess amount of them will be excreted in the urine.^16^ However, fat-soluble vitamins, can be stored in greasy tissues.^17^ Moreover, there are several techniques, such as chromatography and capillary electrophoresis to isolate and purify vitamins.^15^ Biological functions of vitamins are included in Table 1.

**Table 1:** Biological functions of vitamins

**Vitamin**	**Source **	**Recommended dietary allowance (per day)**	**Some of functions in the body**	**Reference**
A	Retinoic acid: Animal tissues Carotenoids: Production by plants, fungi, algae, and bacteria	600-800 (μg retinol activity equivalents)	Anti‐aging, Skin therapy, cytokine modulation, antioxidant, melonocyte function modulation, sunscreen effect, role in vision, bone growth, reproduction, and function of the immune system	^7,18,19^
D	D_2_: Plant products such as mushrooms D_3_: liver, fatty fish, fish oils, egg yolks and human skin by exposure to UV radiation	14000 IU	Skeletal functions including, calcium absorption and bone health, inhibition of diabetes, cancer, and cardiovascular diseases, prevention of postmenopausal osteoporosis, therapy of inflammatory, cell proliferation modulation	^20-22^
K	K_1 _(phylloquinone): Green, leafy vegetables like spinach, collards, and broccoli, and soybean oil and canola oil. K_2_ (menaquinones): Animal/bacteria-derived products	90-120 mg	Activation of particular proteins in atherosclerosis prevention and bone metabolism, procoagulant	^23^
E	In leafy vegetables and fortified cereals	15 mg	Anti-inflammatory activity, Treatment of cancers and cardiovascular disorders, antioxidant, supporting eyes and neurological function, inhibition of platelet coagulation	^24,25^
B_12_	Meat and fish, dairy products, cheese, egg white, and boiled haddock	2.4 μg	Vital co-enzyme for growth of cells, DNA synthesis, nerve cell maintenance	^7,13,26-28^
C	Fruits and vegetables, such as papaya, mango, kiwi, spinach, tomato. lettuce, strawberry, peppers potatoes, broccoli, lemons, cabbage, peas, pears, brussels cauliflower, sprouts, meat organs (heart liver, and kidney), seafood, chicken, and pork	500 mg	Anti-inflammatory and depigmenting effects	^29,30^

## Characterization and role of vitamins in the body

###  Vitamin B

 The B family vitamins include B1 (thiamine), B2 (riboflavin), B3 (niacin), B4 (Choline), B5 (pantothenic acid), B6 (pyridoxine), B8 (biotin), B9 (folate), and B12 (cobalamin).^31^ Mammals cannot synthesize B vitamins on their own; thus, they need to take them up in sufficient amounts from dietary or microbial sources, such as the intestinal microbiota. Although the majority of them are produced by plants, yeasts, and bacteria, they can be found in animal‐derived foods such as eggs, meat, and dairy, through plant consumption by mammals.^11^ Some main sources of B vitamins are broccoli, bananas, potatoes, dates, spinach, asparagus, nuts, figs, and dairy products.^15^ Plants are not able to produce vitamin B12; however, the bacteria located in the ruminants foregut or humans colon can produce this vitamin.^32^ According to the WHO reports, vitamin B12 deficiency will probably be the most prevalent malnutrition problem in the future.^33^

 The B vitamins aim in digestion, boosting immune system and metabolism, and repairing cells.^15^ Vitamins B have an important role as cofactor of enzymes in all tissues, through numerous biochemical pathways. They can enhance the function of the nervous and immune systems,^34^ regulate metabolisms, and improve the cell division and growth. Most of these B vitamins are essential bioactive compounds which are dependent on the diet supply, excluding niacin that can likewise be produced from tryptophan. Vitamin B deficiencies can be frequently seen in elderly, children, pregnant women, vegetarians, and patients with gastrointestinal disorders.^35^ A higher risk of mood and behavioral disorders, increased serum homocysteine levels, and heart diseases were recently connected to vitamin B deficiencies.^36^ B vitamins, specifically B9, B12, and B6 are involved in removing homocysteine from the body, pertaining to the dementia via direct vascular or neurotoxic mechanisms.^37^ For proper neuronal performances, having an adequate quantity of folic acid, vitamin B12, and vitamin B6 is essential. These vitamins possess critical roles in donation of methyl group for synthesizing lipids, proteins, nucleic acids, hormones, and neurotransmitters. However, their deficiencies have been reported to be related to an increased risk of dementia, neurodevelopmental, and psychiatric diseases. Moreover, improper absorption, function, and metabolism of such vitamins are attributed to gene polymorphisms related to the increased occurrence of cognitive disorders.^38^

###  Vitamin C

 Vitamin C, also known as ascorbate, is an essential vitamin broadly distributed in many tissues. This nutrient is plentiful in fruits, vegetables, and animal livers. Vitamin C is comprised of two molecular forms: the oxidized form (dehydroascorbic acid) and the reduced form (ascorbic acid). This vitamin is principal for the physiological performance of the nervous system and antioxidative functions of body through reducing lipid peroxidation, scavenging free radicals, and inhibiting oxidative stress. Furthermore, it participates in several non-oxidative stress processes, such as production of cholesterol, collagen, carnitine, amino acids, catecholamines, and some hormones.^12^ Vitamin C is necessary for the function of two dioxygenase enzymes responsible for the biosynthesis of carnitine, an important cofactor that transports long-chain fatty acids into the mitochondria. Therefore, it has an effect on generating energy via beta-oxidation.^39,40^ Vitamin C is involved in biochemical reactions catalyzed by monooxygenases, dioxygenases and mixed function oxygenases. A lack of vitamin C hampers the activity of a range of enzymes and may lead to scurvy in humans.^41^

###  Vitamin D

 Vitamin D is classified in to two main groups: ergocalciferol (vitamin D2) and cholecalciferol (vitamin D3), which are different in terms of physicochemical traits, molecular structures, and biological effects.^42^ Vitamin D3 can be mainly found in animal foods. Vitamin D2 is mostly found in some wild mushrooms, in which it is converted from a provitamin called ergosterol. Plants utilized as food may have ergosterol, but it is not transformed to vitamin D2 in nature.^43^ Vitamin D has significant impact on brain function and development, mood regulation, dopamine ontogeny, axonal connectivity, neuronal differentiation, immunological modulation, and transcriptional control of a huge number of genes. Vitamin D has been proved to protect neurons from inflammation damage via clearance of gathered amyloid β.^37^ Its deficiency has been attributed to the pathogenesis of some psychiatric disorders, such as depression and autism spectrum disorder.^44^

###  Vitamin A

 Vitamin A is a collection of organic compounds, including retinoic acid, retinol, retinal, and provitamin A called carotenoids.^13^ Vitamin A is a constituent of the pigment rhodopsin situated in the retina, contributing in visual processes and prevention of blindness. It is also involved in the function of gut microbiota, plasma retinol, CD38 (cluster of differentiation 38), and RORA (Retinoic acid receptor-related orphan receptor alpha4) mRNA.^1^ Insufficient levels of vitamin A can cause decreased CARS (Childhood Autism Rating Scale) score, increased level of serum 5-hydroxytryptamine (5-HT), and reduced development of the central nervous system.^45^ Vitamin A is necessary to maintain epithelial integrity and cellular differentiation, production of red blood cell, as well as increase in body resistance against infections. Severe deficiencies of vitamin A lead to vision issues (xerophthalmia). It has been reported that even moderate deficiency of vitamin A may damage vaccine elicited immunity for some types of vaccines.^13^

###  Vitamin E

 Vitamin E (tocopherols and tocotrienols) exists in all membranes of cells and plasma lipoproteins, particularly in human red blood cells. Vitamin E can protect DNA, fatty acids, and low-density lipoproteins from oxidation. It, also, has a role in biosynthesis of hemoglobin, stabilization of the membranes structure, and modulation of immune responses.^41^ Fine sources of vitamin E are vegetable oils, oil seeds, nuts, cheese, egg yolk, margarine, soya beans, oatmeal, wheat germ, avocados, and green leafy vegetables, etc. Deficiency of vitamin E is rare in humans, though it can be seen in premature infants as well as in people with chronic fat malabsorption, mild anemia, and ataxia.^24^

###  Vitamin K

 Vitamin K exists in two natural forms. Vitamin K_1_ (phylloquinone) is ample in leafy green vegetables, such as lettuce, cabbage, and spinach.^46^ The other natural form, vitamin K_2_ (menaquinone) is predominantly from microbial origin.^47^ Vitamin K_2_ is mostly present in fermented foodstuff such as natto and cheese; however, gut microbiota (*Escherichia coli*, *Mycobacterium phlei*, and *Bacillus subtilis)* is able to produce vitamin K_2, _as well.^48^ There is also a synthetic form of vitamin K, which is known as vitamin K_3_ (Menadione).^49^

 Vitamin K has an important promoting role in controlling the bone formation and blood clotting. Vitamin K deficiency may cause hemorrhagic diseases in babies, in addition to muscle hematomas, postoperative bleeding, and intracranial hemorrhages in grown person.^41^ Some food sources containing vitamin K are liver, meat, egg yolk, whole grain, brussels sprouts, vegetables, parsley, celery, iceberg lettuce, cabbage, peas, asparagus, broccoli, cucumbers, and soya bean.^15^

## Nanoencapsulation

 Generally, encapsulation is defined as a process to incorporate bioactive compounds into another compound named cover material, shielding them from environmental and gastrointestinal conditions.^50^ The coated substances (active agents) are also called as fill, core, or internal phase, whereas the coating substances (carrier agents) are known as shell, membrane, wall material, matrix, capsule, or external phase.^51^

 Encapsulation method is widely used to improve the shelf life and bioavailability of bioactive compounds. Encapsulation of food ingredients within nano-capsules, can elevate the stability and bioavailability of bioactive compounds, thus improving food products quality.^52^ Several food-grade materials such as polysaccharides, lipids, and proteins are used to encapsulate bioactive compounds. However, carbohydrates and proteins are not appropriate for industrial aims because of the utilization of complex chemical materials or heat processing that cannot be completely controlled. On the other hand, lipid-based nanocarriers have advantages such as more loading efficiency, biocompatibility, targeted effect, low toxicity, modified release, and ease of constant production.^53^ In the following sections, a summary of different lipid-based nanocarrier properties is given.

###  Lipid-based nanocarriers for vitamin delivery

 Lipid-based nanocarriers bear excellent functionality in film formation, emulsification, and encapsulation of a variety of substances. These systems are generally categorized into two groups. First group is liquid lipid nanoparticles including nanoemulsions, colloidosomes, nanoliposomes, and multiple nanoemulsions. The second group is solid lipid-based nanocarriers including solid lipid nanoparticles (SLNs), and nanostructured lipid carriers (NLCs).^54^ Some important numbers of such delivery systems are briefly discussed in the following sections.

###  Nanoemulsion

 Nanoemulsions are defined as liquid dispersions with droplet sizes of 50 to 500 nm.^55^ This kind of nanocarriers are formulated by water, oil, and surfactants/biopolymers in several types of single water-in-oil (W/O) or oil-in-water (O/W) nanoemulsions, double water-in-oil-in-water (W/O/W) or oil-in-water-in-oil (O/W/O) nanoemulsions, Pickering nanoemulsions formed by biopolymer nanoparticles, and structural nanoemulsions covered by one or two layers of biopolymer materials.^54^ A detailed information pertained to different nanoemulsion structures can be found in different studies.^56-59^ Oil-in-water nanoemulsions are particularly appropriate for nanoencapsulation and carrying lipophilic vitamins due to their reliable physicochemical stability along with acceptable oral bioavailability.^60^ For example, vitamin D_3_ was encapsulated in O/W nanoemulsions. In this work both *in vitro* and *in vivo *studies demonstrated that the nanoemulsion-based delivery system improves the bioavailability of vitamin D3 absorption.^61^ The capability of W/O/W emulsions to provide an effective delivery system for vitamin B_12_ into skim milk was evaluated and 88.85% encapsulation efficiency was obtained by this W/O/W emulsion.^26^ Nanoemulsions are promising carriers owing to their easy preparation, rapid release traits, and high stability. They can be fabricated to meet the particular requirements needed for certain bioactive compounds.^62^

 Nanoemulsions are appropriate for encapsulating, shielding, and carrying both hydrophilic and lipophilic bioactive compounds. Bioavailability and bioaccessibility of bioactive components encapsulated with nanoemulsions are affected by several parameters, such as the type and the physical state of lipid, the size of nanoemulsions, and the nature of surfactants.^63^ Normally, nanoemulsions are created from GRAS (generally recognized as safe) compounds. To increase the stability and decrease the toxicity, surfactants or co-surfactants such as peptides, proteins, polysaccharides, phospholipids, or nonionic surfactants (Tween and Span) are being used in the structure of nanoemulsions.^62,64^

###  Solid lipid nanoparticles 

 SLNs are often mentioned in texts as the first lipid-based nanocarrier group which were designed at early 1990s as a replacement to emulsions, liposomes, and polymeric nanoparticles.^65^ SLNs are a particular kind of nanoemulsions (with diameter range of 50 to 1000 nm), which are fabricated by substituting the oil phase in an O/W nanoemulsion with a solid lipid or a mixture of solid lipids (such as paraffin, waxes, and triacylglycerol). Contrary to nanoemulsions, lipid droplets in SLN systems have high crystallinity, increasing the stability of encapsulated bioactive and prolonging the release process due to much lower diffusion rate.^18,52,66^

 It has been demonstrated that, poly (vinyl alcohol) films containing SLNs with entrapped α-tocopherol, had a higher control on the release of α-tocopherol compared to the neat films, confirming the more controlled release of bioactive components in this system and the possibility of its usage in active packages for foodstuff conservation.^67^

 Owing to the solid based of SLNs, a sustained release of bioactive compounds can be provided. Nevertheless, the most important issue related to SLNs is their low bioactive compound loading capacity as well as the possibility of expulsion throughout the storage.^68^ For example in a study by Couto et al,^69^ only 12% encapsulation efficiency was observed for vitamin B_2_ loaded within SLNs. However, reaching to a higher loading efficiency have also been reported. In this regard, vitamin A-loaded SLNs were successfully prepared using the hot homogenization method with the help of cetyl alcohol and Gelucire 44/14® as carrier materials, which showed more than 90% entrapment efficiency.^70^

###  Nanostructured lipid carriers 

 To overcome some of the problems related to SLNs such as low encapsulation efficiency, NLCs were suggested as the second generation of SLNs.^71^ Nanostructured lipid carriers are spherical shape particles with mean diameters of 50 nm to 500 nm, constituted of a blend of liquid and solid lipids, dispersed within the aqueous media and stabilized using an external layer of surfactants. The NLCs containing vitamin E have been prepared by medium-chain triglycerides, avocado oil or coconut oil as liquid lipids, stearic acid or beeswax as solid lipids, and some nonionic surfactants.^72,73^ The incorporation of oil within a solid matrix results in the creation of amorphous nanostructures with numerous imperfections inside its matrix, granting NLCs to have a higher bioactive capacity and a lower degree of expulsion through the storage, compared to SLNs.^74^ High encapsulation efficiency related to vitamin E (EE:86.6%)^75^ and Vitamin D3 (EE:90.4%)^76^ has been observed in literatures. Important attributes of NLCs such as size, particle distribution, and stability, depend on the components of NLC and the type of the process that is being used to synthesize the particles.^72^

###  Lipid–polymer hybrid nanoparticle 

 Lipomers, lipid– polymer hybrid nanoparticles, have been developed as promising nanocarriers and have gained considerable interest owing to the corresponding beneficial properties of both polymeric nanoparticles and phospholipid shell. This core-shell kind of nanocarriers, in which a lipid monolayer or a liposomal bilayer envelops the polymeric core, has great structural stability provided by the polymer core rigidity, sustained-release ability, biocompatibility, and surface functionality.^77^ However, due to the complexity of their structure, designing of lipomers needs more works and accuracy. Thus, lipomers have not been developed in the industrial scale. Contrary to other lipid-based nanocarriers, lipomers offer some exclusive features such as the variety in structural components, controlled bioactive release, higher encapsulation, improved stability profile, enhanced cellular uptake.^78^ Unlike SLNs and NLCs which are mostly utilized to encapsulate hydrophobic bioactive compounds, Lipid–polymer hybrid nanoparticles are capable of loading several hydrophilic and hydrophobic bioactive compounds due to the coexistence of polymer and lipid providing different material properties, such as hydrophobicity and water-solubility.^79^ In a research study, a protein-lipid composite lipomers with three layers, including barley protein layer, phospholipid layer, and α-tocopherol layer and an inner aqueous partition was fabricated and successfully entrapped vitamin B_12_ with encapsulation efficiency of 69% and prolonged release behavior in a simulated gastrointestinal medium.^80^ Application of different lipid-based nanocarriers for encapsulation of vitamins is summarized in Table 2.

**Table 2:** Application of different lipid-based nanocarriers for encapsulation of vitamins

**Nanocarrier**	**Synthesizing method**	**Formulation ingredients**	**Vitamin**	**Encapsulation efficiency (%)**	**Reference**
Nano-emulsions	Phase-inversion based hot water dilution	Monegyl caprylic-/capric triglyceride, polyethylene glycol hydroxyl stearate, water	D_3_	Up to 90	^81^
	Sonication	Pea protein isolate, canola oil, ethanol, water	D_3_	93	^21^
Solid lipid nanoparticles (SLNs)	High-speed homogenization/solvent diffusion	Phosphatidylcholine, stearic acid, ethanol, water	E	90	^82^
	microemulsion method using stirring	Glyceryl monostearate, tween 80, butanol, water	E	84	^83^
Nanostructured lipid carriers (NLCs)	Melt-emulsification	Stearic and oleic acids, glyceryl monostearate, beeswax, chitosan, tween 80, water	A	98	^84^
	Homogenization and sonication	Phosphatidylcholine, ascorbic acid, glycerol monostearate, Phosphatidyl serine sodium, chloroform-toluene	D_3_	63	^22^
Lipid-polymer hybrid nanoparticles (LPHNs)	Solvent evaporation emulsification	Poly(lactic-co-glycolic acid), ethyl acetate, poly(vinyl alcohol), cholesterol, phosphatidylcholine, chloroform	D	> 60	^85^

## Nanoliposomes

###  Characteristics of nanoliposomes

 Nanoliposome as a vesicular lipid bilayer nanocarrier, is a developing structure capable of encapsulating the biologically active ingredients, ensuring their safe delivery. In general, there are similar chemical, physical and thermodynamic properties between liposomes and nanoliposomes. However, smaller particle size, which means larger surface-to-volume ratio is the advantage of nanoliposomes over liposomes. This feature provides other benefits such as improved bioavailability, increased solubility, exact targeting, and better controlled release of the encapsulated material.^86^ A clinical comparison study on non-liposomal and liposomal vitamin C, demonstrated that the liposomal encapsulated vitamin C has uniform particle size, and well-organized morphological pattern, providing highly efficient encapsulation resulting in an improved bioavailability.^87^ Nanoliposomes are artificial vesicles of spherical shape with small size that are made of natural non-toxic phospholipids and cholesterol. In these systems, water-soluble drugs are encapsulated in the aqueous core which consists of hydrophilic parts of the phospholipids, and the insoluble agents are entrapped in the hydrophobic domain which contains lipid part of the phospholipids bilayer.^88,89^

 Nanoliposomes have different structural sizes and shapes based on environmental circumstances, their constituents, and their production techniques.^90^ Liposomes can be categorized into five types based on their diameter: (1) multilamellar vesicles - 0.5–5 nm with 5 to 20 lipid bilayers, (2) small unilamellar one lipid bilayer vesicles - 20–200 nm, (3) large unilamellar vesicles with one lipid bilayer - 200 nm, (4) giant unilamellar vesicles with one lipid bilayer - 1 nm, and (5) multi vesicular vesicles with multi lipid bilayer - 1 nm.^91^

**Figure 1:**
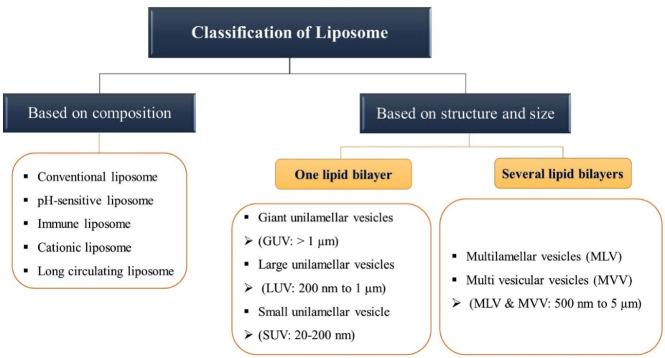
A chart of main types of liposomes and their characteristics.

 Nanoliposomes with a unilamellar state show a balloon-like structure with a simple monolayer, whereas liposomes that are multilamellar have onion like structures consisting of several single-layer cases. Unilamellar liposomes can be categorized as small unilamellar vesicles or large unilamellar vesicles, with diameters below 100 nm and over 100 nm, respectively. Several smaller vesicles are entrapped in a bigger one in multivesicular vesicles.^92^ Another classification of nanoliposomes is: (1) pH-sensitive liposomes: External changes in pH may destroy the lipid composition of liposomes, (2) conventional liposomes: the lipid layer of liposomes is composed of positively and negatively charged phospholipids and cholesterol, which are attached to the aqueous core, (3) immunoliposomes: with antibody molecules on the surface of liposomes, (4) cationic liposomes: with one positive charged lipid or phospholipid in the structure of liposomes, which can interact with nucleic acids and compounds with negative charge, through a simple mixing process, (5) long circulating liposomes: synthetic polymers, glycoproteins, oligosaccharides, and polysaccharides can make a hydrophilic layer on the surface of liposome, which result in the prolonged circulation of the liposomal components in drug delivery systems.^93^ A chart of main types of liposomes and their characteristics is given as Figure 1.

###  Characterization of phospholipids used in the structure of nanoliposomes

 In pharmaceutical and food industries, these systems are formed from surfactants such as phospholipids with relative ratios of hydrophilic-lipophilic balance (HLB) and ideal curvatures about zero. Phospholipids are derived from natural sources such as milk, soy, and egg, so it is safe to use them in food-grade products. In recent years, liposomes were generally produced by phospholipids from eggs and soy^94^; however, milk phospholipids have a higher quantity of glycolipids and sphingolipids in comparison to other phospholipids. Moreover, they have low membrane permeability, thick walls, higher heat transfer, and good stability while storing at 4–35°C temperatures, compared to phospholipids prepared from soybean.^95^ Commercial lecithin is comprised of a blend of several phospholipids and other components such as triglycerides, free fatty acids, and sterols.^92,96^ Phosphatidyl inositol and phosphatidyl ethanolamine can be considered as examples of the most significant phospholipids forming lecithin. Natural lecithins are usually used beside other surfactants in nanoliposome systems due to their HLB of 8, which makes them unsuitable to be used alone for this purpose. Lecithin as zwitterionic surfactant, can have negative, neutral, or positive charges depending on its pH value and electrolyte content. However, these compounds are not stable against oxidation which is probably due to their large amounts of unsaturated fatty acids.^97,98^ Marine phospholipids are other kind of phospholipids which are being used in the structure of nanoliposomes. These phospholipids which contain eicosapentaenoic acid or docosahexaenoic acid, have better resistance to oxidation, high bioavailability, and high nutraceutical properties.^99,100^

###  Characterization of hydrophobic and hydrophilic parts of nanoliposomes 

 Nanoliposomes are able to increase the solubility, bioavailability, and controlled release of the encapsulated material to a larger extent. Hydrophobic bioactive compounds are entrapped in the lipid bilayer of these systems, during their formation process. The compounds inside them can be released when they diffuse through the membranes, or when their membrane is disrupted due to the alterations in temperature or pH, and etc.^101^ These systems can entrap different molecules such as amphiphilics, hydrophilics, and lipophilics which are entrapped into the lipid/water bilayer, into the interior, and within the hydrophobic bilayer, respectively. These heterogenous particles are obtained by placing phospholipids (such as lecithin) in the water or organic solutions and applying enough energy which results in the formation of unilamellar (one bilayer) or multilamellar (series of bilayers) structures.

 While phospholipids can spontaneously turn into unilamellars by applying them in the aqueous mediums, their structure do not show desirable properties and good stability. Suitable production processes should be applied for production of liposomes to obtain appropriate properties such as smaller particle size, high loading capacity, and a proper encapsulation entrapment.^102-104^ Several factors such as temperature, ionic strength, and pH, determine the physicochemical properties of nanoliposomes. Lipid and phospholipid vesicles display low permeability to the entrapped or encapsulated material. Nevertheless, at increased temperatures, a phase transition occurs in them which can have impact on their permeability characteristics. Phospholipids of nanoliposomes have a substantial thermal characteristic in which phase transition (Tc) occurs at temperatures lower than their final melting point (Tm). At Tc, known as gel to liquid crystalline transition temperature, much of the ordered packing arrangement of phospholipid bilayers are being lost, whereas there is an upsurge in its fluidity.^105^ By placing the amphiphilic molecules such as phospholipids in an aqueous phase, they can form aggregated complexes to protect their hydrophobic parts from water molecules; however, they keep their contact with the aqueous phase via the hydrophilic head groups. By presence of enough level of energy, the aggregated phospholipids can organize themselves in the form of closed bilayer vesicles such as liposomes or nanoliposomes. In this way, liposomes can entrap hydrophilic, lipophilic molecules, or lipid soluble compounds such as nutrients, drugs, and certain vitamins.^106,107^ Moreover, there can be other compounds in the structure of nanoliposomes, such as sterols. Sterol incorporation into bilayers of nanoliposome can cause major changes in the properties of these carriers. Cholesterol is the most commonly used sterol in the production of the lipid vesicles. However, it forms bilayer structures by itself. High concentrations of this compound can be incorporated into phospholipid membranes, to modulate the fluidity of the lipid bilayer which results in an increase in the stability of vesicles, and reduced permeability of the lipid membrane to solutes.^108^

 Other sterols have been scarcely used in the structure of nanoliposomes containing vitamins; however, in a study by Amiri et al^10^ the effect of cholesterol and phytosterol (Campesterol) powder was investigated to synthesize a new formulation of nanoliposome for entrapment of vitamin C. The findings showed that the highest stability of vitamin C in a period of 20 days was obtained in phospholipids to campesterol ratio of 75:25. A positive impact of cholesterol substitution with campesterol on control release, encapsulation efficiency, and stability of vitamin C in nanoliposomes was reported. Regardless of current studies, further researches are needed to be done in this field.

###  Methods of nanoliposome preparation

 Several factors such as concentration of the encapsulated material, nature of the medium, physicochemical characteristics of the ingredients, polydispersity, size, and shelf life of the liposome are significant factors to be considered for preparation of liposome or nanoliposome. Preparation method of these systems can vary depending on their lipid composition.

 The preparation methods of liposomes and nanoliposomes usually include the utilization of toxic or non-food grade solvents such as ether solution, ethanol, hexane, chloroform, methanol and detergents, such as alkyl glycoside, cholate, alkyl benzene sulfonates substances and triton X-100 for the solubilization of hydrophobic and/or hydrophilic ingredients.^109,110^ However, there are several techniques to reduce or completely eliminate the use of organic solvents for the formation of liposomes, requiring skilled technical knowledge and high investment for being industrialized such as heating metho.^110^

 Several techniques such as emulsion method, freeze drying double emulsion method, membrane contactor technology, supercritical fluid technology, solvent-nonsolvent method, high-pressure homogenization, dual asymmetric centrifugation, cross-flow filtration with detergent depletion method, thin film hydration, reverse-phase evaporation, solvent/surfactant displacement, heating method, microfluidization, sonication, and extrusion method, are being used for liposome and nanoliposome production.

 In spite of several techniques being used for production of liposomes and nanoliposomes, due to the complexity of most of these methods, their application in the industrial scale is difficult and very challenging. For instance, thin film hydration method followed by sonication was used for the production nanoliposomes to encapsulate different kinds of compounds such as vitamins, previously.^111^ In spite of the production of nano-size liposomes with this method, it is impossible to scale up the process. Some other modifications are needed to overcome the scale up problems of these methods. For instance, to scale up the sonication assisted homogenization process, different parameters should be considered. The cycle of sonication time should be kept constant and a 6 mm tip and 100% amplitude is needed to be used. The power which is delivered to the sample as a result of the number of sonication cycles is also an important parameter, because the sound wave should be amplified on the whole batch volume at the same dimensional properties.^112^ Some of the most important methods of nanoliposome production are summarized below.

## Emulsion and freeze-drying double emulsion method

 In the emulsification method, appropriate surfactants are being used to produce oil-in-water and water-in-oil emulsions. This method is a traditional method; however, freeze-drying double emulsion method is a novel trend, in which cryoprotectants are added to the liposome formulation and W/O/W emulsion will be formed followed by a sterilization step.

## Membrane contactor technology

 In this method, organic phase is placed in the pressurized vessel. A pump directs the aqueous phase to a module and the nitrogen gas is used to push the oil phase into the system. The aqueous phase is subsequently pumped through the membrane contactor module, and liposomes will be formed spontaneously at the time that the lipid and aqueous phases meet. Liposomes prepared using this technology are homogeneous with small particle size and high encapsulation efficiency for lipophilic compounds. Moreover, this method is simple to scale up.^113,114^

## Supercritical fluid technology

 In supercritical anti-solvent method, lipids are readily dissolved in supercritical carbon dioxide and then are precipitated, so that ultrafine particles will be formed. In the supercritical reverse phase evaporation method, which is another supercritical fluid technology, aqueous phase and solid lipid materials are put into a sealed viewing cell and the pressure and temperature are adjusted and then the CO_2_ gas is introduced. CO_2_ is then removed, and liposomes are formed. These methods of liposome preparation do not contain any organic solvents showing their higher advantage compared to conventional methods.^115,116^

## Solvent-nonsolvent method

 In the conventional solvent-nonsolvent method, particles are produced due to the lack of solvent. In this method, firstly solution of the material that is going to be encapsulated is prepared and then the solvent is removed through diafiltration or an evaporation procedure after dispersion in its nonsolvent, and liposomes will be produced due to the lack of solvent.^117^

## Homogenization

 In homogenization method of liposome production, the suspension of drug or any compound to be encapsulated, suddenly passes through a homogenization gap causing high streaming velocity, and in this way liposomes are being produced.^118^ The main drawback of this method is its extremely high operating pressure.^119^

## Dual asymmetric centrifugation

 This method of liposome production includes an additional sample rotation around its own vertical axis which pushes sample toward the center of the centrifuge, while in the conventional centrifugation sample is constantly being pushed outwards. Therefore, two overlaying movements of the materials occur in the centrifugation vial, which provides shear forces and an efficient homogenization for even a concentrated and viscous blend of lipids.^120^ This method is easy to operate and no organic solvent is being used for production of liposomes with small particle size.^114,120^

## Cross-flow filtration detergent depletion method

 One of the production methods of liposomes is based on detergent addition to solubilize lipids which is being removed through next steps. In this technique membrane can still contain a huge amount of detergent which is difficult to remove. Therefore, a technique, which can solve this problem and reduce the preparation time and heterogeneous liposome lamellarity is preferred. Combination of cross-flow technique and conventional detergent depletion method is an economical technique which is used to overcome the problems related to the detergent removal.^121^ This method is consisted of a ﬁltration device, a pump, a starting reservoir, tubing, an integrated rotary slide valve and a manometer for monitoring the pressure of retentate.^114^

## Thin film hydration method

 Thin-film hydration method is one of the simplest methods to form liposomes which is followed by extrusion. In this method by removing the organic solvent in a round-bottom flask a thin lipid film is being made. At first step, heterogenous liposomes are made, then homogeneous small liposomes are obtained after extrusion through polycarbonate membranes.^122^

**Figure 2:**
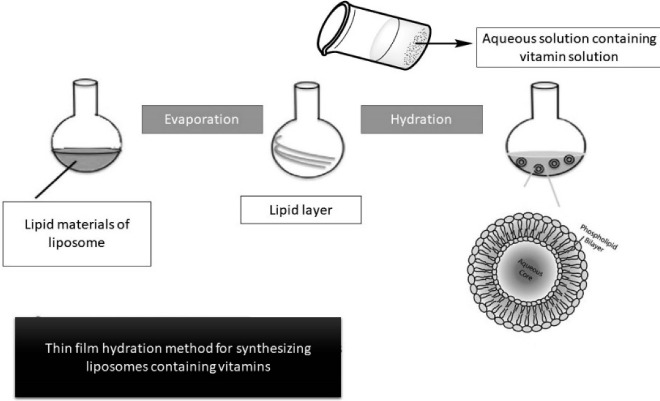
A schematic of thin film hydration method to produce nanoliposomes.

 Schematic thin film hydration method to produce nanoliposomes is illustrated as Figure 2.

## Reverse-phase evaporation method

 In reverse-phase evaporation method, several pure or mixed phospholipids can be used. The solvent is removed by rotary evaporator. At next step by purging nitrogen into system, lipid is dissolved in the organic phase again and the reverse phase vesicles will be made in this step. After this step, the aqueous phase is added which contains materials that are going to be encapsulated, followed by sonication and evaporation of solvent until a gel is formed.^123^

## Solvent/surfactant displacement method

 In solvent/surfactant displacement method, firstly phospholipids are dissolved in an amphiphilic organic solvent, then the mixture is inserted into the aqueous solution (containing surfactant), and the nanoliposomes are created. Sonication and homogenization are also used to decrease the liposomes formed by different methods.^124^

## Heating method

 In this method, high pressures or toxic solvents are not used. All of the compounds including lipids are added to a heating flask and are heated at high temperatures for about 30 min to let lipids are dissolved. Depending on the liposomal compounds, nanovesicles are formed.^105^

## Microfluidization

 Microfluidization is a common type of homogenization method which is widely used in food industry. This technique generates high pressures, directing the flow stream to the impingement area through microchannels. This technique provides acceptable costs for large scale productions and does not include toxic solvents, which favors food regulatory requirements. However, there are three drawbacks for it such as contamination, material loss, and hard scale-up.^125,126^

## Sonication method

 Sonication is a simple way for production of nanoliposomes from liposomes. In this method, hydrated vesicles are treated by a titanium-tipped probe sonicator for a few minutes with determined seconds of on- and off- intervals in a temperature-controlled environment. At this stage, nanoliposomes in the form of small unilamellar vesicles are formed.^105^

## Extrusion method

 In the extrusion method of liposome or nanoliposome preparation, micrometric liposomes are modified to large unilamellar vesicles or nanoliposomes which depends on the pore-size of filters.^127^ Vesicles are extruded by passing through polycarbonate filters with defined pore sizes for several times. At the end, a homogenous sample (nanoliposome) is produced.^105^

###  Advantages and disadvantages of nanoliposomes

 Generally, liposomes and nanoliposomes are systems contributing to the recent food trend. These systems can improve the efficiency of food additives and reduce the amounts of required bioactive compounds. They favor the green chemistry, and they can increase the use of natural compounds instead of synthetic constituents.

 Comparing the lipid based nanocarriers with other encapsulation materials such as carriers based on polymeric compounds, shows that lipid based nanocarriers have distinctive advantages, such as their ability to provide a protective cover for biological compounds which protects them from degradation, their capability of entrapping the compounds with broad range of solubility, and their high potential for industrial production due to the natural food components being used in their formulations, as well as their minimum production costs. In addition, they have good biocompatibility and biodegradability.^128^ According to USFDA (United States Food and Drug Administration) guidance, liposomes are different than drug-lipid complexes, emulsions, and microemulsions. Thus, developing a liposomal product needs suitable certification for its chemistry, production, and constituents, beside bioavailability in human pharmacokinetics and labeling (FDA 2018). Nanoliposomes have several advantageous and useful features in the formulation, application and delivery; however, they have limited drawbacks such as poor encapsulation efficiency, lack of enough parameters for continuous industrial production and stability, excessive cost of food components, and the use of high force pressure, homogenization, and sonication.^129^

 In contrary to micron-sized particles, nanoliposomes have remarkable properties such as being metastable and dilatable with water, without any changes in their size distribution. Moreover, nanoliposomes can encapsulate and liberate two materials with different solubilities such as vitamin C and vitamin E, simultaneously. Nanoliposomes are able to include and deliver both vitamin C and vitamin E to an oxidation site, resulting in a synergistic effect. Lipid based vesicles incorporating two bioactive agents are called bifunctional vesicles.^130-132^ In other study, Chaves et al^133^ showed that it is feasible to produce “co-encapsulated liposome” vesicle containing two curcumin and vitamin D_3, _which is stable over the storage time of more than 40 days.

 One of the most useful characteristics of liposomes and nanoliposomes is their targetability. Directing bioactive molecules to a specific part in which they can apply their optimum efficacy, is very important. An appropriate and directed release enhances the efficacy of bioactive material, certifies optimal dosage, expands the range of their application, and thus increases the cost-effectiveness of the product.^102,134^ Moreover, nanoliposomes provide a delivery option for multiple drug molecules at the same time.^135,136^

 Structural appearance and stability of the nanoliposomes are highly associated with the technology adopted for their synthesis. Several problems related to physicochemical properties and stability of conventional nanoliposomes have been reported. Studies showed that liposomes might not be able to provide good physical and chemical stability resulting in low encapsulation efficiency, lipid oxidation, aggregation of vesicles or fast release of bioactive compounds.^137,138^ Moreover, rapid clearance of most of the liposomes from circulation by the reticuloendothelial system, and fast leakage of water-soluble drugs under improper storage conditions, are some other problems related to these formulations.^139,140^

 Thus, modification of nanoliposomes using methods such as addition of charged substances on their surface, coating their surfaces with polymers, or using some drying techniques after their formation, have emerged as efficient approaches to boost their physicochemical properties and stability.^141-143^ In addition to the formation of biopolymer-associated liposomes, another promising method to improve the bioactivity, stability, and bioavailability of liposomes is to accomplish additional processes, referred to as “post-processing techniques”, to the aqueous liposome. Some common methods of post-processing include spray drying, freeze drying, and spray freeze drying.^144^

## Important properties of vitamin-loaded nanoliposomes

###  Particle size, PDI and zeta potential of nanoliposomes

 Generally, the average particle size, and polydispersity index (PDI) are parameters that are of high importance in liposomes and nanoliposomes. PDI, shows the quality of the formulated system with regard to the size distribution. The suitable application of nanocarriers including nanoliposomes for a specific route of drug administration is highly dependent on their average particle size, PDI and size stability. Size variations of nano sized systems are of high importance in the formulation of nanocarriers and to achieve ideal results, constant and narrow size distribution should be considered. It should be considered that for nanocarriers, size stability is more important, in comparison to micro size systems, and this is due to their larger specific surface area.^145^ In a study on the liposomal formulation of glucosamine and vitamin D, it was shown that the encapsulated liposomes have average particle size of 840 nm with great stability.^146^

 PDI is known as a parameter indicating uniformity of nanocarriers. When this parameter is more than 1, it means that different sizes of particles exist per volume unite in the solution. Particle aggregation increases the PDI, so factors causing aggregation can affect PDI. Studies on lipid-based nanoparticles have shown that several factors such as surfactant type and its concentration, instrumental conditions, and lipids used in their formulations can have impact on PDI and particle size.^147^

 A study on encapsulation of vitamin E in nanoliposome revealed that the formulation of liposomes by hydrophobic stabilizers such as gamma oryzanol and lauric acid leads to an increase in the particle size. Though, hydrophilic stabilizer such as PEG 400 did not have significant effect on mean size. In addition, it was shown that by using hydrophobic stabilizers, particle size distribution (PDI) decreases in colloidal system which indicates their stabilizing effect during storage time while PEG had only a slight effect on PDI.^148^

 Results of a study showed that particle sizes of vitamin C-folic acid liposomes and chitosan coated vitamin C- folic acid liposomes are 138.58 nm and 249.13 nm, respectively. Also the reported PDIs were 0.18 and 0.31, respectively, indicating the similarity in the size of nanoliposomes.^149^ El Adawy et al^150^ could produce nanoliposomes encapsulating ascorbic acid with particle size of 421.6 nm and PDI of 0.539, showing narrow and homogenous particle size distributions.

 Zeta potential is known as the electrokinetic potential between particles which are dispersed in a solution. This parameter shows the surface electrical charges of particles as well as their stability. Zeta potential and the size distribution are both measured by dynamic light scattering (DLS) technique. A large positive or negative zeta potential of particles present in the suspension results in less tendency of them to aggregate, so they will have higher stability.^151^ To broaden the zeta potential of nanoliposomes, providing stronger repulsion, some researchers use charged substances such as biopolymers as an extra layer covering the surface of nanoliposomes. The results of a study on chitosan-coated nanoliposomes of vitamin D3 showed that coating the nanoliposomes with 0.01% (w/v) chitosan can improve favorable properties of nanoliposomes. The PDI of this covered nanoliposome was close to the monodisperse distribution. Moreover, the increase in size and zeta potential verified the interaction of chitosan and liposome, showing a successful coverage.^152^ The increase in zeta potential is due to the more adsorbed cationic polymers on the surface of nanoliposomes. Since chitosan possess a high positive charge, the adsorption of chitosan seems to increase the density of positive charge, resulting in a more positive zeta potential.

###  Encapsulation efficiency

 Encapsulation of vitamins into nanoliposomes provides many advantages, which has been highlighted in this review. One of the most significant benefits of encapsulation of vitamins within liposomes is improving their bioavailability in the human body. The encapsulation efficiency (EE) can be considered as the most important factor to determine the capability of nanoliposomes to encapsulate bioactive compounds. Encapsulation efficiency can also be stated as trapping efficiency, incorporation efficiency, or encapsulation percentage.^153^ The EE depends on the composition of nanoliposomes and the characteristics and concentration of the encapsulated bioactive compound.^154^ Dalmoro,Bochicchio ^152^ reported that the EE of D3 and K_2_-loaded in cholesterol and phosphatidylcholine nanoliposomes was 88.4% and 94.7%, respectively. The chitosan coverage for each nanoliposomal formulation of both vitamins, led to up to 98% increase in the entrapment efficiency. Lee, Park^155^ have reported that multilamellar vesicle nanoliposomes of retinol, composed of L-α-phosphatidylcholine (PC) and 10% sterol (w/w), could increase the EE of retinol up to 99%.

 Since higher EE is correlated with longer shelf-life and better stability of the nanocarrier, it is always favorable to elevate the EE by selecting the best possible mixture of core-wall ratio, processing steps such as drying methods, homogenization process, and other processing variables.^156^ The EE of an entrapped bioactive in a nanoliposome depends on its partition coefficient and polarity. If loaded ingredients are hydrophobic in nature, they reside in the hydrocarbon chain of nanoliposome. However, if loaded bioactive compounds are polar, they are likely to be positioned in the aqueous core or next to the water–lipid interface, adjacent to the nanoliposome polar head groups.^157^ Overall, the EE of lipophilic materials is usually higher than hydrophilic ones in nanoliposomes, since they can be tightly positioned in the membrane.^158^ For instance, in a recent study, two vitamins were simultaneously co-encapsulated within synthesized nanoliposomes: vitamin C as a hydrophilic and vitamin E as a lipophilic bioactive. Vitamin E, and vitamin C displayed an average EE of 95.1% and 77.8 %, respectively. Thus, vitamin E demonstrated the highest EE, which can be attributed to its high lipid affinity.^159^ A summarized application of nanoliposome for encapsulation of vitamins is given in Table 3.

**Table 3:** Application of nanoliposome for encapsulation of vitamins

**Formulation ingredients**			**Fabrication method**	**Coating compounds**	**Encapsulated vitamin**	**Optimum specifications**			**Nano-encapsulation efficiency (%)**	**References**
**Emulsifier**	**Sterol**	**Organic solvent**				**Size (nm)**	**PDI**	**Zeta potential (mv)**		
MFGM phospholipid concentrate, SFPC	Cholesterol	SC-CO_2_	Vent-RESS	-	C/E	MFGM-based: 533 SFPC-based: 761	-	MFGM-based: −57 SFPC-based: −37	MFGM-based: C: 65 E: 77 SFPC-based: C: 72 E: 88	^160^
Phosphatidylcholine	Cholesterol	SC-CO_2_	Vent-RESS	-	C/E	580	-	−40	C: 60 E: 88	^161^
Phosphatidylcholine	Cholesterol	SC-CO_2_	Vent-RESS		C/E	2130	-	−30	C: 78 E: 95	^159^
Soybean L-α- phosphatidylcholine	Cholesterol	Ethanol	Novel simil-microfluidic	-	D_3_/K_2_/E	D_3_: 87 K_2_: 145 E: 118	D_3_: 0.40 K_2_: 0.31 E: 0.38	D_3_: −38 K_2_: −36 E: −27	D_3_: 88 K_2_: 95 E: 93	^162^
Soybean phosphatidylcholine, rapeseed lecithin	-	-	-	-	C	180	-	-	-	^163^
Phosphatidylcholine	-	Ethanol	Thin-film evaporation	-	C	Below 100	-	−40	66	^164^
Egg yolk phosphatidylcholine	Cholesterol	Dichloromethane: Ethanol	Ethanol injection	-	C	229	0.32	−26	71	^165^
Marine phospholipid	-	Ethanol	Thin-film evaporation	-	C	237	0.20	−39	52	^128^
Phosphatidylcholine	-	-	Thermal method with stirring, homogenization, and sonication	Milk protein concentrate, modified starch, gum Arabic, and maltodextrin	D	120	-	-	74	^166^
Egg yolk Phosphatidylcholine	-	-	Thin-film dispersion	Maltodextrin, gum Arabic, modified starch, and whey protein	D_3_	140	-	-	-	^167^
Phosphatidylcholine	Cholesterol	Ethanol	Thin-film evaporation	β-Lac	A	-	-	-	With β-Lac: 56 Without β-Lac: 51	^168^
Sesame phospholipids	-	Chloroform	Thin layer formation, ultra-sonication and hydration in phosphate buffer solution	-	C	-	-	-	80	^169^
Soybean L-α-Phosphatidylcholine	Cholesterol	Ethanol	Microfluidic method	CS	D_3_/K_2_	D_3_: 87 K_2_:145	D_3_: 0.24 K_2_: 0.28	D_3_: –38 K_2_: –36	D_3_: 88 K_2_: 95	_152_
Soybean phosphatidylcholine	Cholesterol	Ethanol, Tween 80	Ethanol injection	-	C/FA	**C-Lip: **145 **FA-Lip:** 109	**C-Lip: **0.17 **FA-Lip: **0.15	**C-Lip: **25 **FA-Lip: **−28	**C-Lip = **32 **FA-Lip = **49	_149_
Soybean phosphatidylcholine	Cholesterol	Ethanol, Tween 80	Ethanol injection	CS	C/FA	**CFA-Lip: **139 **CS-CFA-Lip: **249	**CFA-Lip: **0.18 **CS-CFA-Lip: **0.31	**CFA-Lip: **34 **CS-CFA-Lip: **+25	**CFA-Lip:** **C = **35 **FA = **64 **CS-CFA-Lip:** **C = **81 **FA = **88	_170_
Phospholipid	-	Ethanol	Microfluidic method	Xanthan and guar gums	D_3_	1131	0.20	−49	91	_171_
Hydrated Phosphatidylcholine	-	-	Thermal method with sonication treatment	Gamma oryzanol, lauric acid, polyethylene glycol 400	E	**With vitamin E:** 111 to 138 **Without vitamin E:** 122 to 153	0.19 to 0.38	−37 to −78	>75	_148_
DPPC	-	Chloroform	Thin film hydration	-	Ascorbic Acid	422	0.54	-	23	_150_
Soybean phosphatidylcholine	Cholesterol	Absolute ether	Reversed-phase evaporation	-	Thiamine (TH)/ Niacin (NA)	**NA-Lip:** 136 **TH-Lip:** 126	**NA-Lip:** 0.28 **TH-Lip:** 0.22	**-**	**NA-Lip:** 30 **TH-Lip:** 30	_172_
Soybean L-α- phosphatidylcholine	Cholesterol	Chloroform	Thin film hydration	Sorbitol	C	3050	**-**	**-**	52	_173_
Soybean phosphatidylcholine	Cholesterol	Anhydrous ethanol and chloroform	EI, FH, MFH, and RP	-	Pyridoxine hydrochloride (B_6_)	154 with RP method	0.18 with RP method	**-**	44 with MFH method	_174_
Soy phosphatidylcholine	Cholesterol	Chloroform	Thin-film hydration	-	E	181	-	−22	-	_175_

PDI, Poly dispersity index; MFGM, Milk fat globule membrane; SFPC, Sunflower phosphatidylcholine; SC-CO2, Supercritical carbon dioxide; β-Lac, β-lactoglobulin; FA, Folic acid; DPPC, 1,2 Dipalmitoyl-sn-glycero-3-phosphatidylcholine; Vent-RESS, Venturi-based rapid expansion of supercritical solutions; EI, ethanol injection; FH, film hydration; MFH, Modified film hydration; RP, Reverse-phase evaporation; CS, Chitosan; PDI, poly dispersity index; Lip, liposome.

###  Biocompatibility

 Biocompatibility shows the ability of nanoliposomes to apply their proposed function without having any negative effect on the targeted tissues. There are several studies mentioning the biocompatibility and biodegradability of nanoliposomes loaded with bioactive compounds. Liposomes are comprised of natural lipids which are biodegradable, biocompatible, and less immunogenic. Preliminary skin toxicity study of oleic acid liposomes showed that no epidermal cell apoptosis occurs in the skin that is treated with these liposomes, indicating good biocompatibility of them with mouse skin.^176^ In a study by Al-Ogaidi, chitosan and alginate which are biocompatible compounds were used to manufacture biodegradable and biocompatible nanoliposomes of vitamin C.^140^

 There are several compounds which can be used in the formulation of nanoliposomes to provide biocompatible liposomes. For instance, chitosan has been used in combination with sodium tripolyphosphate to provide core-shell of nanoliposomes for vitamin E encapsulation.^177,178^ Chitosan provides an outer hydrophilic barrier by formation of a coating layer onto membrane, which can prevent the interaction between liposomes, increase their drug delivery efficiency, improve their structural properties, and biocompatibility.^179^

 In other study, L-α-phosphatidylcholine which packages with 1α,25(OH)2D3, and 1,2-distearoyl-sn-glycero-3-phosphoethanolamine-N-[amino-(polyethylene glycol)2000], were used to produce non-toxic and biocompatible vitamin D nanoliposomes.^180^

 Moreover, in spite of biocompatibility properties of liposomes, these formulations could decrease the toxicity of antimicrobial agent which are potentially toxic.^176^

## Controlled release

 The nanoencapsulation systems compared to the direct application of bioactive compounds provide better functional properties such as controlled-release, and higher bioavailability due to their high surface area, in comparison to large particles.^181,182^ Encapsulation provides a surrounding for bioactive compounds or drugs, which protects them against environmental stresses and controls their release over time.^183,184^ Several studies have shown the controlled release of different bioactive compounds using nanoliposomes.^185,186^ Hydrophilic and hydrophobic compounds can simultaneously and efficiently be trapped in the phospholipids bilayer membrane structure of nanoliposome through various physical and chemical interactios.^187^ Therefore, nanoliposome is able to extend the residence time of compounds or drugs in the ambient, causing the controlled *in vivo* release of these compounds, which enables the activity of compounds for a longer time.^188^

 The control release of loaded compounds in nanoliposomes can be improved by doing some modifications on the surface of conventional nanoliposomes.^189^ Researchers have reported that the surface decoration of nanoliposomes of neohesperidin by chitosan and pectin loaded, improve the controlled release of this compound.^190^ Chitosan nanoparticles were shown to improve the controlled release vitamin C in several studies.^191,192^ In a study by Liu et al,^193^ the release behavior of vitamin C was observed for the chitosan and alginate coated nanoliposomes. It was indicated that addition of these polymers onto the surface of anionic nanoliposomes improves the control release of vitamin C during 90 days of storage at 4°C, by a steric barrier on the surface.

## Bioavailability of nanoliposomes containing vitamins

 Bioavailability is a key parameter of pharmacokinetic, which states the proportion of a bioactive compound or a drug, administered through any non-vascular route which reaches to the systemic circulation.^194^ A number of studies have indicated the increased bioavailability of vitamins by loading them in nanoliposomes. A review of recent literature shows a growing trend to increase the bioavailability of vitamins by loading them in nanoliposomes. Łukawski et al^195^ conducted a study with the aim of comparing the profiles of serum concentration of vitamin C in 20 healthy volunteers, after the oral administration either as an aqueous solution or as a liposomal suspension. Their results showed that in the nanoliposomal vitamin C treatment group, C_max_ of vitamin gets to higher values compared to the vitamin C solution treatment group (303 µµ compared to 180 µµ). Moreover, for nanoliposomal formulation, the maximum vitamin C blood concentration delay time (T_max_) is longer by about 1 h in comparison to the solution (T_max_=180 min vs. 96 min). The incremented half-life (t_1/2_>6 h vs. t_1/2_=4 h) and increased AUC (81 570 µµ^*^ min vs. 45 330 µµ^*^ min) shows that loading vitamin C in nanoliposomes improve the bioavailability of this vitamin. The same result was reported by Davis et al^196^ who compared the bioavailability of vitamin C in free and liposomal forms.

 Moreover, in another study conducted on the bioavailability of encapsulated and free vitamin C, Gopi and Balakrishnan^197^ indicated that nanoliposomal vitamin C is 1.77 times more bioavailable compared to the free vitamin C. The nanoliposomal vitamin C showed higher values of C_max_ (5.23 vs 2.17 mg/dL), AUC_0-t_ (55.86 vs 31.53 mg.h/dL), and AUC_0-∞_ (78.90 vs 57.12 mg.h/dL), compared to the aqueous solution of vitamin C.

## Conclusion

 In this review, the application of nanoliposomes to encapsulate vitamins was investigated. Regarding to vitamin encapsulation, nanoliposomes are known as the most used lipid based nanocarriers. One of the most principal reasons for the high use of nanoliposomes to deliver vitamins is related to their ability to encapsulate both hydrophilic and hydrophobic vitamins as well as the ease of their production in industrial scale. However, the quality of produced nanoliposomes is very important. The fabrication of a high quality nanoliposome containing vitamins mainly depends on the production method, utilized materials, and characteristics of liposomes such as particle size, PDI, zeta potential, controlled release, and encapsulation efficiency. Lack of attention to these parameters, while producing nanoliposomes, can easily lead to the fabrication of systems with several drawbacks such as fast release, deposition, low stability, and imperfect protection of vitamins. Recent trends demonstrate that researchers are interested to synthesize nanoliposomes with the highest encapsulation efficiency of vitamins, thus many of the recently published studies have reported reaching to the higher than 70% encapsulation efficiency for different vitamins.

 In addition to inherent properties of nanoliposomes, the bioavailability of loaded vitamins into nanoliposomes is another significant factor. Increased bioavailability can be considered as the final goal for the application of nanoliposomes to cover different vitamins. As mentioned in this review, there are clear recent evidences showing that nanoliposomes are able to improve the bioavailability of vitamin C. Moreover, based on our knowledge, some variables still need more investigations. For instance, the replacement of cholesterol with other sterols and their effects on different factors of vitamin-loaded nanoliposomes require more studies. Moreover, interaction of vitamins with other compounds that are incorporated into nanoliposomes, and their effects on several parameters of nanoliposomes such as stability, zeta potential, and encapsulation efficiency should be investigated.

## Acknowledgments

 This study is related to the project NO. 1399/62182 From Student Research Committee, Shahid Beheshti University of Medical Sciences,Tehran,Iran. We also appreciate the “Student Research Committee” and “Research & Technology Chancellor” in Shahid Beheshti University of Medical Sciences for their financial support of this study.

## Author Contributions

**Conceptualization: **MasoudAman Mohammadi, Parastou Farshi.

**Data curation:** Parastou Farshi.

**Formal Analysis: **MasoudAman Mohammadi.

**Funding acquisition:**No one.

**Investigation: **Azam Ahmadi, Parisa Ahmadi.

**Methodology: **MasoudAman Mohammadi, Parastou Farshi.

**Project administration:** Marjan Ghorbano, Seyede Marzieh Hosseni.

**Resources:** Masoud Aman Mohammadi.

**Software: **Masoud Aman Mohammadi.

**Supervision: **Marjan Ghorbani.

**Validation: **Mohammad Yousefi.

**Visualization:** Mohammad Yousefi.

**Writing – original draft: **Masoud Aman Mohammadi, Parastou Farshi, Azam Ahmadi, Parisa Ahmadi.

**Writing – review & editing:**Marjan Ghorbani, Seyede Marzieh Hosseini.

## Ethical Issues

 Not applicable.

## Conflict of Interest

 The authors declare that they have no competing interests.
